# Readaptation of the Vestibulo-Ocular Reflex Relieves the Mal De Debarquement Syndrome

**DOI:** 10.3389/fneur.2014.00124

**Published:** 2014-07-15

**Authors:** Mingjia Dai, Bernard Cohen, Eric Smouha, Catherine Cho

**Affiliations:** ^1^Department of Neurology, Icahn School of Medicine at Mount Sinai, New York, NY, USA; ^2^Department of Otolaryngology, Icahn School of Medicine at Mount Sinai, New York, NY, USA

**Keywords:** optokinetic, posturography, Fukuda stepping test, velocity storage, rocking, swaying, bobbing

## Abstract

The mal de debarquement syndrome (MdDS), a continuous feeling of swaying, rocking, and/or bobbing, generally follows travel on the sea. The associated symptoms cause considerable distress. The underlying neural mechanisms are unknown, and to date there have been no effective treatments for this condition. Results in monkeys and humans suggested that MdDS was caused by maladaptation of the vestibulo-ocular reflex (VOR) to roll of the head during rotation. We studied 24 subjects with persistent MdDS (3 males, 21 females; 19.1 ± 33 months). Physical findings included body oscillation at 0.2 Hz, oscillating vertical nystagmus when the head was rolled from side-to-side in darkness, and unilateral rotation during the Fukuda stepping test. We posited that the maladapted rocking and the physical symptoms could be diminished or extinguished by readapting the VOR. Subjects were treated by rolling the head from side-to-side while watching a rotating full-field visual stimulus. Seventeen of the 24 subjects had a complete or substantial recovery on average for approximately 1 year. Six were initially better, but the symptoms recurred. One subject did not respond to treatment. Thus, readaptation of the VOR has led to a cure or substantial improvement in 70% of the subjects with MdDS. We conclude that the adaptive processes associated with roll-while-rotating are responsible for producing MdDS, and that the symptoms can be reduced or resolved by readapting the VOR.

## Introduction

Mal de debarquement, characterized by sensations of swaying, rocking, and/or bobbing, commonly occurs after voyages on the sea (1). Of 236 sailors, 73% had this postadaptive syndrome for up to 24 h (mean = 2.6 h) (2). Some of these individuals do not recover, and the symptoms can persist for months or years. This prolongation of symptoms has been labeled the mal de debarquement syndrome (MdDS) (3–5), and it is associated with many other disturbing symptoms, including disorientation, impaired cognition, fatigue, ataxia, insomnia, headache, anxiety, and depression (5, 6). MdDS disappears temporarily when riding in a car or during travel on water, but the symptoms return after the ride. The diagnosis is made on the basis of the symptoms. Vestibular function is normal, and treatment is generally ineffective.

Experiments in subhuman primates provided insights into the cause of the disorder (7). Monkeys were rolled from side-to-side while rotating in darkness for several hours. Before conditioning, the eyes only torted as the animals were oscillated in roll. Afterwards, the monkeys developed vertical and horizontal nystagmus in addition to the ocular torsion. The vertical nystagmus oscillated as they rolled from side-to-side, with upward slow phases when the animals were on one side and downward slow phases on the other side. They also had horizontal nystagmus. The alteration in the eye movements indicated that the vestibulo-ocular reflex (VOR) had been maladapted. This only occurred in monkeys with long VOR time constants, and was not present when the VOR time constant was close to that of the cupula [4.5 s; Ref. (7)]. This implies that the maladaptation of the VOR depended on the central integrative mechanism in the vestibular system, i.e., velocity storage (8, 9).

Similar maladaptation was produced in humans in NASA space flight experiments (10). Subjects rode in a slow rotating room for long periods during which they rolled their heads at timed intervals. Afterwards, they developed oscillating vertical nystagmus on head roll. From the monkey and human data, we posited that MdDS was due to maladaptation of the VOR, and that this maladaptation could be reversed by inducing opposite responses with full-field optokinetic stimulation to act against the maladapted components. This hypothesis was tested in this report.

## Materials and Methods

### Subjects and enrollment

Twenty-four subjects (3 males, 21 females, ages 28–64, mean 43.0 ± 8.8 years; Table 1) were recruited through the MdDS foundation and from medical referrals. For inclusion, the MdDS subjects had to have continuous rocking, swaying, and/or bobbing that began shortly after exposure to a voyage on water or in the air and that persisted for months or years. The symptoms were debilitating and were not relieved by any medications or other medical treatments. Their symptoms were temporarily better during car rides or travel on water but returned after the rides were terminated. Exclusion criteria included a history of head and neck trauma, vestibular dysfunction, or major neurological or vascular disorders. Subjects signed an informed consent approved by the Institutional Review Board at the Icahn School of Medicine at Mount Sinai.

**Table 1:** **Subjects, physical findings, and treatment effects**.

Subject no.	Sex/age (years)	Duration (months)	Vertic Nyst	Fuk St test	Posturog freq (Hz)	Arm freq (Hz)	Score PreTr	Score PostTr	Follow-up (months)
S1	F/35	1	+	−	0.2		10	0	18
S2*	M/50	9	−	−	0.13		10	1	8
S3	F/29	5	+	+	0.3	0.4	10	1.5	7
S4	F/64	11	+	−		0.2	7	1.5	18
S5	F/57	20.4	−	−	0.3	0.3	6	1.5	16
S6	F/40	9	+	+	0.1	0.1	7	2.5	16
S7	M/42	36	+	−		0.45	5	0	17
S8	F/50	36	−	−	0.23	0.2	8	8	
S9	F/42	96		+	0.18	0.14	6	6.5	
S10	F/41	5	−	+		0.17	6	0	16
S11	F/46	144	−	+	0.13	0.16	8	8	
S12	F/44	2	+	+	0.27	0.2	8	8	
S13	F/45	8		−		0.18	8	2.5	16
S14	F/40	5		+	0.18	0.16	8	8	
S15	M/45	15.6	−	+		0.23	5	0	13
S16	F/46	4		+	0.23		6	0	10
S17	F/40	24	−	−	0.3		5	5	
S18	F/49	6		+	0.11	0.11	5	0	8
S19	F/28	2		−	0.23	0.19	8	1	7
S20	F/51	6	+	+		0.16	5	0.5	12
S21	F/40	3	−	+	0.14	0.23	6	6	
S22	F/28	1.5		−		0.22	7	0	6
S23	F/29	5		+	0.17	0.31	6	0.5	5
S24	F/52	4	−	+		0.18	6	0	4
Mean ± SD	43 ± 8.8	19.1 ± 33	7 of 16	14 of 24	0.2 ± 0.1	0.2 ± 1	6.8 ± 0.7	0.7 ± 0.9	11.5 ± 0.2

*+ Is present or positive; − is not present or negative; blank box indicates not tested; duration, of MdDS before treatment; Vertic Nyst, oscillating vertical nystagmus; Fuk St test, Fukuda stepping test. Posturog Freq is frequency of fore/aft rocking from posturography. Arm Freq is frequency of arm movement mimicking internally perceived rocking. Score PreTr is the pre-treatment score. Score PostTr is rating at the fourth month after treatment. Follow-up, for those treated successfully*.

S2* results show effects of second treatment after relapse.

### Oscillation frequencies and posturography

In order to reverse the maladapted VOR, several physical signs were determined. These included the frequency of rocking and the direction of the optokinetic stimulus used for treatment. Not every MdDS subject had actual physical oscillations, e.g., those with bobbing, but they all felt the oscillations internally. Frequencies of these internally perceived movements were registered with a 3-D accelerometer attached to the wrist. Subjects were seated with eyes closed. They rested their elbow on a padded plate and made “forward–backward” arm movements synchronously with the internally perceived sense of rocking. The predominant frequency of the oscillations was determined with Fourier analysis. Measurements of these arm movements were obtained in 20 out of 24 subjects (Table 1). The oscillation frequency from arm movements was used as the rocking frequency for the subsequent test and treatment. If not available, the frequency from posturography was used.

Posturography measurements were taken in 16 MdDS subjects before treatment and in 11 MdDS subjects after the first treatment session. Posturography was also done in 11 normal subjects to provide a basis for comparison with the effects of treatment. Eight MdDS subjects were not tested with posturography because they had little or no observable rocking. On examination, the tested subjects stood quietly on a Nintendo Wii force plate with their feet 28 cm apart for 2 min with eyes open and for 2 min with eyes closed. The fore–aft rocking and medial–lateral sway were registered and the data were recorded on a PC through Bluetooth communication. Fourier analysis of the rocking data from the self-indication and rocking/swaying data from posturography were used to determine the frequency spectra of the recorded traces. These frequencies were then used to direct the head roll during treatment (see below).

### Nystagmus

**Figure 1:**
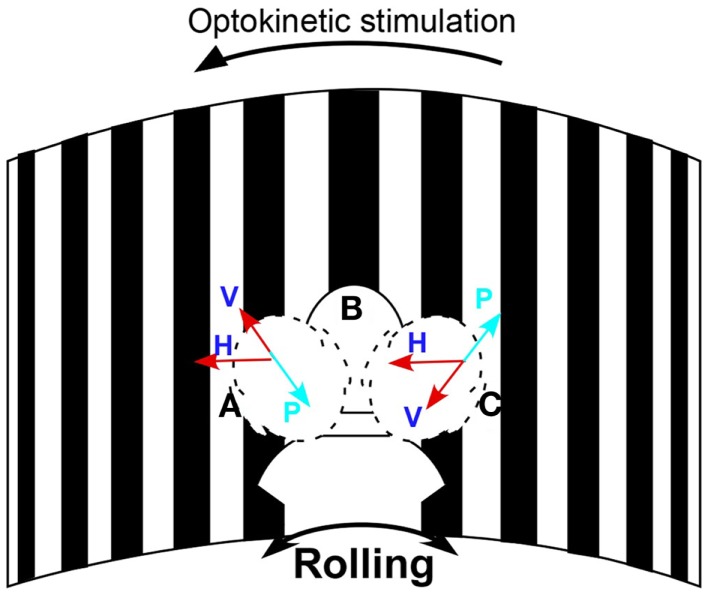
**Schematic diagram of the treatment paradigm**. The subject’s head was passively rolled at her rocking frequency while watching the stripes moving to the left to act against the maladapted VOR component to the right. H is the velocity of the horizontal optokinetic stimulus; V is the component of the optokinetic stimulus in the head sagittal plane; P represents the maladapted vertical component; Head positions: A, on the left, B, in the center, and C, on the right. Arrows show the direction of the horizontal and vertical slow phase velocity.

Eye movements were measured in darkness using video-oculography at 30 frames/s (sensitivity 2°/s) to determine if subjects had vertical nystagmus. The head was rolled 20° from side-to-side by the examiner at the frequency of rocking determined by self-indication. A metronome was used to direct the head movements at the appropriate frequency. A 3-D accelerometer detected head position and movement. Vertical eye positions were extracted from the eye images with a cubic spline interpolation and were differentiated to yield eye velocity. If present, the slow phases were upward when the head was rolled to one side and downward when the head was rolled to the other side. The direction of the oscillating vertical nystagmus was used to determine the direction of the treating optokinetic stimulus. For example, if the slow phase velocity was upward when the head was to the right and downward with the head on the left, the optokinetic stimulus was given to the left to counteract the vertical components (Figure 1).

### Fukuda stepping test

Rotation in the stepping test can give an indication of a sided imbalance (11), and it was used to indicate the direction of the treating optokinetic stimulus. Subjects marched in place with eyes closed and arms outstretched for 1 min twice. Rotation of >20° to the same side on both trials indicated a horizontal imbalance in the VOR; if they rotated to the left, the treating visual stimulus was rotated to the right.

In case there was no vertical nystagmus or rotation in the stepping test, the direction of the treating optokinetic stimulus was determined from the subjective rotation. In this instance, the visual field would rotate to the same side as their perceived rotation. If none of these indications were present, an arbitrary optokinetic rotation was given briefly. If the direction of the visual stimulus was not correct, there could be an increase in the subject’s symptoms or no response, and the direction was reversed.

### Treatment

After determination of the direction of the optokinetic stimulus and the rocking frequency, subjects were seated in a circular room (diameter, 6 ft) that had an optokinetic stimulator that projected 1.4° black and white stripes on the wall that filled the subject’s field of vision (Figure 1). During treatment, the stripes were rotated at a constant velocity (Figure 1). Rotation of the drum elicited optokinetic nystagmus, and it also produced a sense of self-rotation (circular vection). The direction of the sensed rotation was opposite to that of the direction of rotation of the visual field. The subjects’ heads were rolled ±20° at their rocking frequency by the examiner while the subject watched the rotation of the stripes. A metronome tone directed the rocking frequency. The velocity of the optokinetic stimulus was initially 10°/s, but could be altered according to the subject’s response. Each session lasted for 3–5 min. Subjects were treated over 1 week. They had one to eight treatments per day for up to 5 days. When the severity of their MdDS was reduced by ≥50%, they were not necessarily treated further. That is, some subjects wanted to get additional treatment to quench any remaining symptoms. Subjects were followed for 4 months to 2.5 years. The average follow-up was 11.6 months (Table 1).

### Outcome measures

The severity of MdDS was rated on a subjective scale from 0 (asymptomatic) to 10 (most severe). Ratings were given at the end of the initial treatment in whole numbers or in half integers and were verified by telephone at various time up to one year. Ratings were primarily based on the amount of rocking, swaying, and bobbing, but also included a general estimate of the level of malaise. Complete recovery was defined as freedom from all symptoms. A substantial remission was a reduction in the level of symptomatology to between 1 and 3 from 6 to 9. Posturography was also used to verify changes in body sway and rocking.

### Statistics

A Student’s *t*-test was used to compare the treatment effects.

## Results

None of the subjects had overt vestibular or oculomotor dysfunction based on electronystagmography using rotation, caloric stimulation, ocular pursuit, and optokinetic stimulation. The results are summarized in Table 1. Twenty-four subjects with a history of MdDS from 1 month to 12 years (mean 19.1 ± 33.3 months) were studied. Twenty-one had the symptoms after exposure to travel on water. Two developed the symptoms after flight and one after riding on a motion simulator. The baseline severity of MdDS before the treatment was 6.9 ± 1.6.

### Rocking frequency

**Figure 2:**
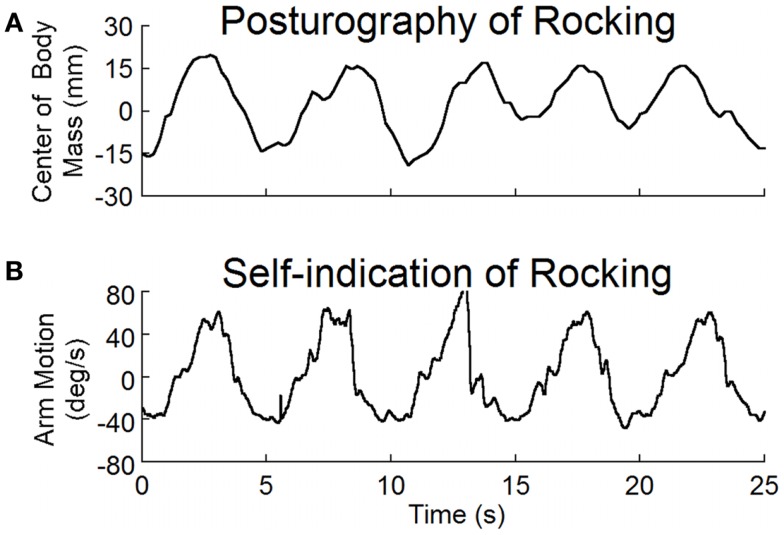
**(A)** Posturographic recordings of body rocking, and **(B)** recordings of arm movements at the perceived frequency of rocking. Both body movement and arm movement were at the same frequency of about 0.2 Hz.

The average frequency determined by both posturography and by self-indication was 0.2 ± 0.1 Hz (Table 1). There was no difference in the frequencies determined by these two methods (*p* = 0.5, *N* = 11, paired *t*-test). Examples of recordings of posturography and self-indication are shown in Figure 2. The frequencies of the arm movements were the same for this subject with eyes open or closed, or when standing or sitting. Similar verification in other subjects justified the use of the arm movements to estimate the rocking frequencies in subjects without overt rocking.

### Oscillating vertical nystagmus

**Figure 3:**
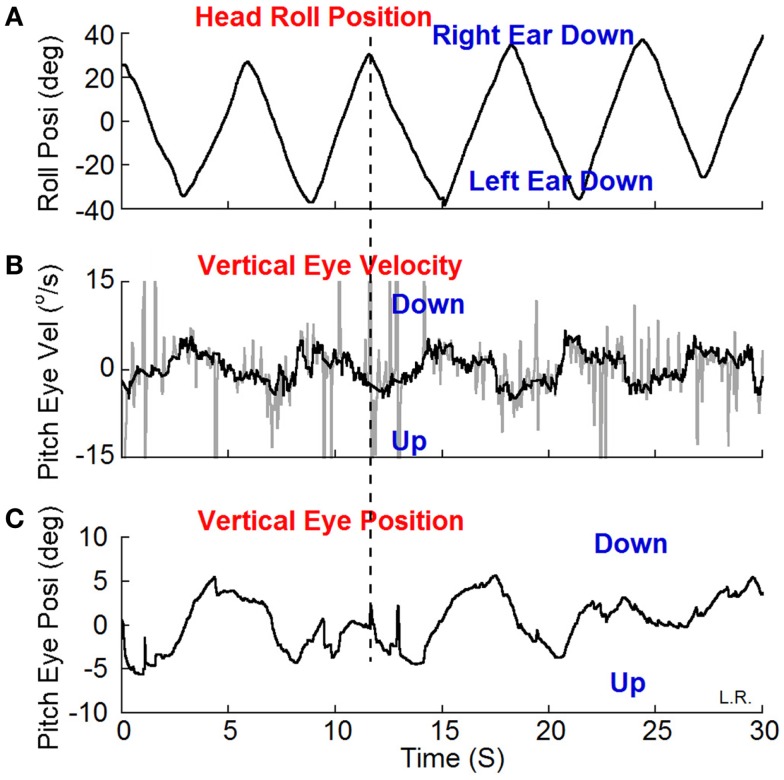
**Vertical (pitch) eye position (C) and velocity (B) induced by head roll (A) at about 0.2 Hz in darkness**. **(B)** The dark traces show the slow phase eye velocity and the gray traces the quick phases, blinks and fluttering of the eyelid. The peak velocity of the vertical eye oscillations was about ±4°/s estimated by a sine function approximation. The vertical dash line shows the relationship between the head position in roll and slow phase vertical eye velocity and position. The slow phase eye velocity was upward when the head was rolled to the right and down when the head was rolled to the left.

Weak oscillating vertical-nystagmus was present in 7 out of 16 subjects (Table 1).A typical example is shown in Figure 3. Head roll of ±20° at 0.2 Hz elicited the nystagmus in darkness. When the head was rolled to the right the slow phase eye velocity was upward (Figure 3B, Up) with a phase of 197° and a peak velocity of 4°/s by a sine function approximation. When the head was rolled to the left, the slow phase velocity was downward. Other subjects had a reversed response. The relationship of vertical nystagmus and head position was the same as that in monkeys (7).

### Fukuda stepping test

All subjects were tested with the Fukuda stepping test. Fourteen rotated from 20° to 45° clockwise or counter clockwise in 1 min (Table 1). The subjects were tested at least twice and the results were the same. Two subjects had weak horizontal spontaneous nystagmus (≤2°/s). The slow phases of the nystagmus were in the direction of a positive Fukuda stepping test and were opposite to the direction of treatment in these individuals.

### Rocking and swaying

**Figure 4:**
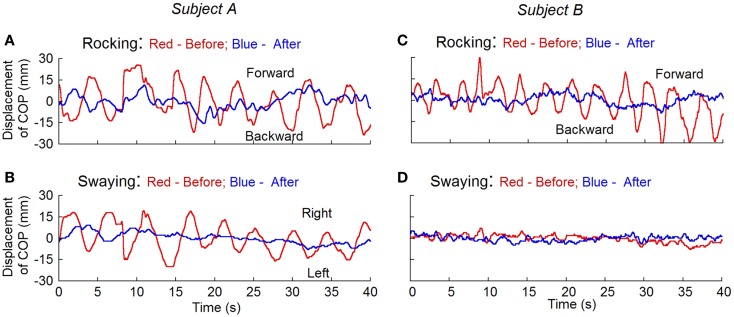
**Measurement of the displacement of center of pressure (COP) before (red traces) and after treatment (blue traces) for rocking and swaying with posturography in two subjects**. Subject A had both rocking and swaying whereas subject B mainly had rocking. After treatment, the rhythmic oscillations present before the treatment disappeared.

Posturography was studied in 11 subjects before and after the treatment and in 11 normal (control) subjects. The root mean square (RMS) of the displacement of the center of body mass (RMS) (12) was calculated for the magnitude of rocking. To remove the offset, the traces were displayed with the subtraction of the mean value from the recorded data that were taken 10 s after the subjects’ eyes were closed. Before the treatment, the RMS rocking was 8.3 ± 3.0 mm and the sway was 5.7 ± 1.9 mm in the MdDS subjects. In all MdDS subjects, the movements were predominantly fore/aft, i.e., rocking. In five subjects, there was also significant sway that combined with the rocking to form a circular progression of the trunk around the stationary feet. The subjects could transiently stop the movements, but the movements returned shortly thereafter. These values were significantly reduced after the treatment to 3.0 ± 1.3 mm (*p* = 0.001, *N* = 11) and 2.8 ± 0.6 mm (*p* = 0.01, *N* = 11), respectively. The RMS displacement in normal subjects was 4.0 ± 1.5 mm for rocking and 3.2 ± 1.0 mm for swaying. Values of rocking and swaying in the MdDS subjects before treatment were also significantly different from the values in the control subjects (*p* = 0.001 and 0.003, respectively). There was no difference between the amplitudes of rocking and swaying in the treated subjects and the controls (*p* = 0.2 and 0.08, respectively). None of our normal subjects had a distinctive dominant frequency of oscillation, recognized by visual inspection of the time series of raw data and of frequency spectra of position. This is consistent with the findings of others (13). Figure 4 shows the recordings of rocking and swaying before (red lines) and after the treatment (blue lines) in two subjects. Treatment substantially reduced the rocking in both.

#### Direction of the treating optokinetic stimulus

In the seven subjects with oscillating vertical nystagmus, the direction of the optokinetic stimulus was determined from the vertical nystagmus (four left and three right). In 10 subjects’ the direction was determined from the stepping test (8 left and 2 right). In three subjects, the direction was taken from the subjective perception of rotation (one left and two right). In four subjects, the direction was determined arbitrarily (two left and two right). Altogether, there were 15 subjects in whom the optokinetic stimulus rotated to the left and 9 to the right.

#### Treatment outcome

Seventeen out of 24 subjects (70%) became asymptomatic (10 subjects; PostTr of 0 or 0.5) or had a substantial remission (7 subjects; PostTr of 1–2.5) for 4 months or longer. The pre-treatment severity in these 17 subjects was 6.8 ± 1.7 versus 0.7 ± 0.8 after treatment (*p* < 0.01, *N* = 17).

Four of eight subjects traveling a long distance home by car and one subject traveling by air had a reversion of their symptoms after the return trip, although initially they had relief of their symptoms after treatment. One additional subject had a reversion of symptoms after working on a large computer screen. One subject did not respond to the treatment. One treated subject experienced a relapse of symptoms after 6 months. The subject was then successfully retreated and reported levels of 0–1. Five subjects developed severe unilateral, pulsing headaches. In all, the headache subsided and the MdDS did not return.

## Discussion

The MdDS, a debilitating condition, was successfully treated in this study. Based on findings in the monkey (7) and in NASA spaceflight experiments (10), we postulated that the syndrome was caused by maladaptation of the VOR. This maladaptation had added vertical and horizontal components to ocular torsion induced by head roll and body oscillations at a frequency that was centered around 0.2 Hz. Using a full-field visual stimulus, given while the head was rolled at the frequency of the subjects’ rocking, the MdDS was reversed in 23 out of 24 subjects, although the treatment effects regressed in 6. In the other 17 (70%), the MdDS was cured or substantially reduced for prolonged periods (mean follow-up = 11.6 months).

A unique part of this study was to demonstrate that the MdDS does indeed have physical findings, although the findings were not present in every subject. Seven out of 16 had weak oscillating vertical nystagmus, 14 out of 24 had turning on the Fukuda Stepping Test, and all of the subjects had either actual or internally felt oscillations. A striking finding was that the oscillations, which were commonly present in most of the subjects, were close to a common frequency, i.e., 0.2 Hz (1 oscillation/5 s). Each of these symptoms and signs disappeared after the subjects were successfully treated. The rocking and swaying of the MdDS subjects were significantly larger than the oscillations of a normal, healthy control group (*p* = 0.001 and 0.003). After treatment, these differences disappeared (*p* = 0.2 and 0.08). Thus, while the subjects were not studied with a larger control group, the treatments caused significant differences in their physical findings, and brought them closer to the status of normal subjects.

The successful treatment supports the hypothesis that there had been readaptation of the VOR, caused by roll-while-rotating. The roll and rotation most often took place on sea voyages, probably when vessels sailed obliquely to the direction of the waves and wind. The roll could be induced by the ships movement on the waves and the rotation was presumably produced by bringing the ship back on course (14). Similar maladaptation has also been produced during flight, probably by causing oscillation of the plane while banking [Dutch roll, (15)].

A parallel experiment in primates using pitch-while-rotating (7) did not produce similar adaptation although the semicircular canals were continuously activated. Pitch-while-rotating was also attempted in three MdDS subjects, but it was ineffective in reducing their symptoms (unpublished data). Furthermore, a subject who complained of MdDS after ferry rides was advised to face in the direction of the waves during the ride. This minimized roll and substantially reduced the post-ride MdDS. It suggests that a way to reduce post sail-MdDS would be to hold the craft as close to the direction of the wind and waves as possible.

How and where the adaptation of the VOR took place and where the 0.2 Hz oscillations originated is of considerable interest since it is also likely to shed light on the mechanisms that are responsible for producing the more common sensation of mal de debarquement, which has similar symptoms, but is of much shorter duration (1, 2). Several findings indicate that the VOR adaptation had involved velocity storage in the vestibular system, which integrates activity from the semicircular canals and the visual system, and is largely responsible for the dominant time constant of the VOR (8, 9, 16). This mechanism is also responsible for producing vection, i.e., a sense of rotation from visual motion (17, 18), and vection was present in each of the subjects with MdDS in response to full-field visual motion. The adaptation of the VOR was directly related to the amplitude of the dominant time constant in the monkey and did not occur in monkeys with short VOR time constants (7). From this, we would predict that neither mal de debarquement, nor MdDS, would occur in people with very short VOR time constants.

Treatment of the subjects was done with a full-field visual stimulus, which engages pretectal visual pathways. These pathways carry the input to velocity storage in the vestibular nuclei and to the inferior olive and cerebellum (19, 20), where the adaptation is likely to have occurred and the frequency of rocking, swaying, and bobbing are likely to have been generated (21). The observation that full-field visual training induces oscillations in eye and head movement in other animal models exhibiting robust velocity storage strongly supports our hypothesis and the use of a corrective visual waveform (22).

In summary, MdDS is a rare and debilitating disease. Using findings from monkeys, the current work provides a plausible hypothesis to explain the cause of the condition. Treatment based on this hypothesis produced significant relief in this group of subjects over a follow-up time of approximately 1 year. Many questions remain, such as why the condition is relieved temporarily by car, train, or plane rides, why the process strikes women more often than men, and exactly how and where the process(es) that produce the rocking, swaying, and bobbing are induced in the nervous system. One unexpected benefit is that by understanding the adaptive mechanisms that produced the MdDS, it may be possible to understand the cause of mal de debarquement.

## Author Contributions

Dr. Mingjia Dai was responsible for the study, data processing and manuscript. Dr. Bernard Cohen was an advisor of the project. He discussed the cases and wrote most of the manuscript. Drs. Eric Smouha and Catherine Cho sent the patients, discussed the cases and treatment strategy with Dr. Mingjia Dai.

## Conflict of Interest Statement

The authors declare that the research was conducted in the absence of any commercial or financial relationships that could be construed as a potential conflict of interest.

## Abbreviations

MdDS, mal de debarquement syndrome; rocking, forward/backward oscillation; swaying, side-to-side oscillation; bobbing, up/down oscillation; RMS, root mean square; maladaptation, unusual adaptation of the VOR so that there are abnormal eye and body responses when the VOR is activated; COP, center of pressure.
